# Poisson pre-processing of nonstationary photonic signals: Signals with equality between mean and variance

**DOI:** 10.1371/journal.pone.0188622

**Published:** 2017-12-07

**Authors:** Michaela Poplová, Pavel Sovka, Michal Cifra

**Affiliations:** 1 Institute of Photonics and Electronics, the Czech Academy of Sciences, Chaberská 57, 182 51, Prague 8, Czechia; 2 Faculty of Electrical Engineering, Czech Technical University in Prague, Technická 2, 166 27, Prague 6, Czechia; University of Pécs Medical School, HUNGARY

## Abstract

Photonic signals are broadly exploited in communication and sensing and they typically exhibit Poisson-like statistics. In a common scenario where the intensity of the photonic signals is low and one needs to remove a nonstationary trend of the signals for any further analysis, one faces an obstacle: due to the dependence between the mean and variance typical for a Poisson-like process, information about the trend remains in the variance even after the trend has been subtracted, possibly yielding artifactual results in further analyses. Commonly available detrending or normalizing methods cannot cope with this issue. To alleviate this issue we developed a suitable pre-processing method for the signals that originate from a Poisson-like process. In this paper, a Poisson pre-processing method for nonstationary time series with Poisson distribution is developed and tested on computer-generated model data and experimental data of chemiluminescence from human neutrophils and mung seeds. The presented method transforms a nonstationary Poisson signal into a stationary signal with a Poisson distribution while preserving the type of photocount distribution and phase-space structure of the signal. The importance of the suggested pre-processing method is shown in Fano factor and Hurst exponent analysis of both computer-generated model signals and experimental photonic signals. It is demonstrated that our pre-processing method is superior to standard detrending-based methods whenever further signal analysis is sensitive to variance of the signal.

## Introduction

Photonic signals lie at the heart of modern sensing methods used for environmental protection [1], food safety [2], and early detection of biomarkers of diseases such as cancer [3] and neurodegenerative diseases [4]. Analysis and processing of photonic signals and their statistical properties are also crucial in quantum optics and communication technologies [5]. Hence, robust signal analysis and processing of photonic signals and their statistical properties are essential for exploiting photonic technologies to their limits.

Advanced analysis of photonic signals extends well beyond mere detection of the mean intensities or optical wavelength spectra of photon signals; photocount distributions [6, 7], correlation analyses [8], and fractal/chaos-based signal analysis techniques [9] are required to fully exploit the information carried by the photonic signals under study. Most of these methods of signal analysis inherently assume stationary signals. If the signal contains an unwanted trend that is unrelated to the analyzed process, detrending methods exploiting the trend removal estimated by smoothing (moving average, exponential or Gaussian approximation) or robust smoothing [10] have to be applied to make a signal stationary in order to prevent artifactual findings. While the detrending is typically a straightforward task for many types of common non-photonic signals, the story is far more complicated for photonic signals. Due to their intrinsic quantum nature they are naturally non-negative integer signals and typically exhibit a Poisson-like photocount statistics [11], which brings a coupling between the mean and variance of the signal [12]. This coupling poses a problem for the currently available signal pre-processing and detrending methods that find and subtract the mean of the signal: the information about the mean still remains in the variance of the signal. These issues are especially pronounced for the signals of low intensity that occur when one strives for high optical spectral resolution or when the generation process itself is very weak, which is the case for the signals from advanced photonics methods such as those employing Raman-scattering [13] or electro/bio/chemiluminescence analysis [14–17]. While most pre-processing methods applied on Poisson and Poisson-like signals perform variance stabilization, *e*.*g*. Anscombe or Bartlett transforms [18–21], which is employed in signal denoising, there are no methods for proper detrending and stationarization of Poisson signals up to our knowledge.

In this paper, we develop a method for proper pre-processing of nonstationary signals originating from any process with a Poisson distribution. We demonstrate the superiority of our method compared to the detrending methods on both computer-generated model signals and experimental luminescence signals.

## Poisson signals

Photonic signals are non-negative integers with Poisson-like distribution. In such distribution, the signal mean and variance are interconnected. Therefore we first summarize the statistical properties of signals with Poisson distribution

$$\begin{array}{c} f\left(k;\lambda \right)=Pr\left(X=k\right)=\frac{\lambda^{k}}{k!}\cdot e^{-\lambda },\;k=0,1,2,... \end{array}$$ (1)

which is a discrete probability distribution, where λ is the average number of events in a specified interval such as time, distance, area or volume. The random variable *X* = 0, 1, 2… is a non-negative integer number. The cumulative probability function is

$$\begin{array}{c} F_{p}\left(k;\lambda \right)=\sum_{i=0}^{k}\frac{\lambda^{i}\cdot e^{-\lambda }}{i!}. \end{array}$$ (2)

When λ is sufficiently high, the Poisson distribution can be approximated by a normal distribution [22]:

$$\begin{array}{c} \hat{F_{p}}\left(k;\lambda \right)=\frac{1}{\sqrt{2\lambda \pi }}\int_{-\infty }^{k}e^{-\frac{{\left(k-\lambda \right)}^{2}}{2\lambda }}du. \end{array}$$ (3)

For example when λ = 40, the maximum of the absolute error,

$$\begin{array}{c} \epsilon =\underset{k}{\text{max}}|F_{p}\left(k;\lambda \right)-\hat{F_{p}}\left(k;\lambda \right)| \end{array}$$ (4)

will be approximately 0.01.

The Poisson distribution has a special property:

$$\begin{array}{c} \lambda =E(X)=\text{Var}(X); \end{array}$$ (5)

that is, the mean is equal to its variance. This property is corrupted if common pre-processing methods are used such as detrending procedures (which find the trend using smoothing or robust smoothing methods), data normalization such as min-max [23] or decimal scaling [23], or both detrending and normalization procedures together. Alternatively, the method based on the Z-score [22, 23],

$$\begin{array}{c} Z=\frac{X-\mu }{\sigma }, \end{array}$$ (6)

where *μ* is the mean and *σ* is the standard deviation of the value of a random variable *X*, is often used. In the next text we will use a simplified notation for random processes (signals). Typically the symbol *X*(*ϵ*_*l*_, *n*) is used where *ϵ*_*l*_ represents *l*—th realization of the random signal and *n* is the time instant of the discrete-time random signal. Instead of this symbol we are going to use a simplified notation *x*[*n*]. Then expected value *E*[*x*[*n*]] = ∑_*i*_ *p*_*i*_ *x*_*i*_[*n*] represents the ensemble average of the discrete-time random signal at the time instant *n*. Similarly, $Var[x[n]]=\sum_{i}p_{i}\;x_{i}^{2}\left[n\right]$ represents the variance of the random process at the time instant *n* evaluated over the ensemble of realizations.

**Fig 1:**
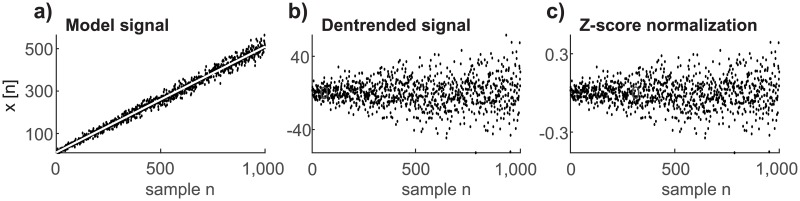
Nonstationary poisson signal preserves its variance after detrending. a) A model signal with Poisson distribution, linearly increasing trend *t*[*n*] (white line) according to equation *t*[*n*] = 0.5 ⋅ *n* + 10 for each sample of signal *n* = 1, 2…1000; b) the detrended signal is created by subtraction of the trend from the model signal; c) the pre-processed model signal after Z-score normalization.

Experimental photonic data are naturally discrete in time, and therefore we use a discrete-time approach to describe our method and signals. Fig 1 illustrates the problems of detrending and normalization (6) of the signal with a Poisson distribution. Fig 1a depicts the original nonstationary signal with a Poisson distribution. Each sample of the signal can be considered as one realization of a random process with a Poisson distribution with its parameter λ evolving in time such that λ = λ[*n*]. One can see that the variance and mean are closely interconnected. An increasing time-varying mean value (trend, *t*[*n*] = λ[*n*] = E(*x*[*n*])) causes increasing variance, as suggested in (5). The detrended signal *x*_*d*_[*n*] = *x*[*n*] − *t*[*n*] still has a growing variance that contains information about the increasing trend of the original signal (Fig 1b). Z-score normalization ensures both signal detrending and normalization by the average variance (scale change), but information about the time-varying mean value is still preserved in the form of nonstationary growing variance (see Fig 1c). Thus the relation between the mean and variance after detrending or Z-score normalization is corrupted:

$$\begin{array}{c} t\left[n\right]=\lambda \left[n\right]=0\neq Var\left(x\left[n\right]\right)=\sigma^{2}\left[n\right]. \end{array}$$ (7)

Moreover, the samples of the resulting signal *t*[*n*] are not integers anymore. The other two normalization methods (min-max transformation and decimal scaling) mentioned earlier give the same results as the Z-score normalization.

**Fig 2:**
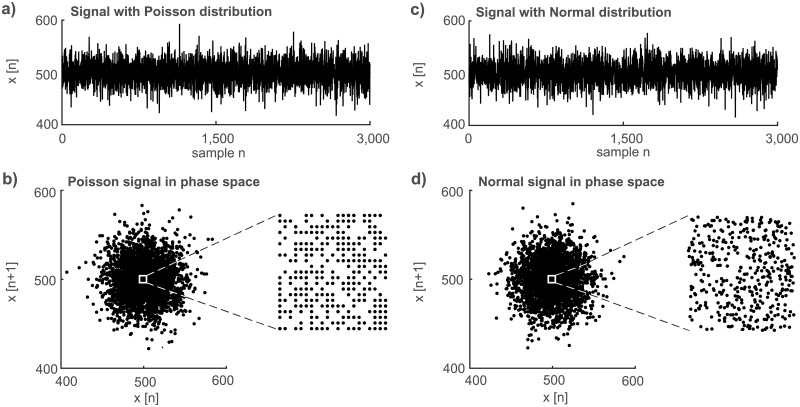
Phase space plot shows a marked difference of signals originating from poisson vs. normal distribution. a) Time waveform of a model signal with Poisson distribution (λ = 500, *n* = 3000); b) its depiction in the phase space and a close-up view of its central part. c) Time waveform of a model signal with normal distribution (*μ* = 500; $\sigma =\sqrt{500}$, *n* = 3000); d) its depiction in the phase space and a close-up view of its central part.

The second inherent property of a random process (signal) with Poisson distribution is a rectangular grid in the phase space (*x*[*n*], *x*[*n* + 1]) depicted as a close-up view in Fig 2b. This property follows from the fact that the Poisson distribution allows only integer numbers while most of the random processes, for example signals with a normal distribution, form an irregular random grid in this phase space (Fig 2d, close-up). This grid irregularity is caused by the lack of real numbers in the respective realization of the random signal. It is worth emphasizing that it is necessary to use the zoomed-in view of the cluster because the shapes of the whole clusters of the two random processes (Fig 2b:Poisson distribution; Fig 2d:normal distribution) as well as the time signal wave-forms are similar (Fig 2a and 2c).

## Materials and methods

### Poisson pre-processing

The suggested Poisson pre-processing (PP) method is based on Z-score normalization (6). Z-score transformation is originally applied in order to normalize a random variable with normal distribution [24] and is frequently used for the signal detrending and signal variance normalization [22]. The Z-score method standardizes the signal into a signal with zero mean and a standard deviation equal to one. This type of transformation of a random variable with normal distribution preserves the type of distribution [22]. It changes only its mean and variance. Eq (6) can be modified for Poisson random variable

$$\begin{array}{c} S=\frac{X-\lambda }{\sqrt{\lambda }}. \end{array}$$ (8)

For a discrete-time nonstationary signal with a Poisson distribution Eq (8) can be rewritten into

$$\begin{array}{c} s\left[n\right]=\frac{x[n]-t[n]}{\sqrt{t[n]}}, \end{array}$$ (9)

where *x*[*n*] ≥ 0 represents signal integer samples, and *t*[*n*] is the trend of the signal for each time instant (instead of *μ* in (6)). The assumption is that one sample *x*[*n*] can be thought as one realization of an integer random variable with Poisson distribution (1) for each time instant, with λ = *t*[*n*]. Therefore, according to (5), the trend *t*[*n*] is also equal to the variance Var(*x*[*n*]), and the standard deviation *σ* from (6) is replaced by the square root of the variance $\sqrt{t[n]}$. Consequently, (9) standardizes the detrended signal (*x*[*n*] − *t*[*n*]) according to its dynamically changing standard deviation. The standardized signal *s*[*n*] has zero mean E[*s*[*n*]] = *μ*_*s*_[*n*] = 0 and unity variance E[*s*^2^[*n*]] = *σ*_*s*_[*n*] = 1 for all time instants. Our goal is to detrend the signal *x*[*n*] while preserving the relation between mean and variance which is typical for Poisson distribution. To reach this goal it is necessary to recover a positive integer samples of the signal *p*(*n*) with a Poisson distribution, the following transformation has to be used:

$$\begin{array}{c} p\left[n\right]=\lfloor \left(\sqrt{t^{\prime }}\cdot s\left[n\right]+t^{\prime }\right\rfloor , \end{array}$$ (10)

where

$$\begin{array}{c} t^{\prime }\geq \left|\underset{n}{\text{min}}\left(x\left[n\right]-t\left[n\right]\right)\right| \end{array}$$ (11)

for all *n* = 1, 2, …*N*, where *N* is equal to the number of signal samples. The symbol âŒŠ*X*âŒ‹ represents the integer part of a variable *X* and the symbol |*X*| represents the absolute value of a variable X. The whole algorithm consists of a detrending and normalizing part (9) and a restoring part (10). The numerator of (9) provides a detrending signal *x*[*n*] so that the trend of the signal *x*′[*n*] = *x*[*n*] − *t*[*n*] is zero. The denominator of (9) decreases (normalizes) the variance of the signal $x^{\prime \prime }\left[n\right]=\frac{x^{\prime }\left[n\right]}{\sqrt{t[n]}}$ to the value of Var(*x*″) = 1. Operations in both the numerator and denominator clearly break the relation between the signal mean and variance, $\mu_{s}\left[n\right]\neq \sigma_{s}^{2}\left[n\right]$. To restore the relation between the signal mean and the variance, (10) has to be realized. The second term of the right side of this equation ensures that the signal mean is nonzero, *μ*_*p*_[*n*] > 0, so that all samples are non-negative *p*[*n*] ≥ 0. The first term of the right side of this equation ensures that the signal variance is equal to the signal mean $\mu_{p}\left[n\right]=\sigma_{p}^{2}\left[n\right]$. The last operation yields the integer part of the result. Converting numbers to non-negative integers performed by Eq (9) ensures that resulting signal samples represent a Poisson signal, that is they are non-negative numbers with *μ*_*p*_[*n*] = *σ*^2^[*n*], *n* = 0, 1, …

As described above, the suggested pre-processing procedure should change only the mean and variance of the measured signals but not their type of distribution. Moreover this procedure ensures that the mean of the signal equals the variance and that samples of the signal are non-negative integers. Both features are connected with a Poisson distribution.

### Estimation of trend

**Fig 3:**
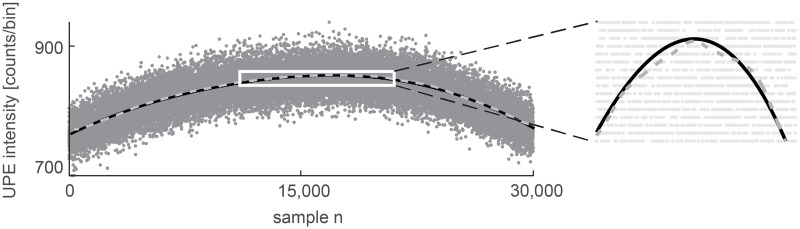
Estimation of trend. Experimental nonstationary signal from neutrophils (grey dots) and their trend obtained by the two-Gaussian-fitting method (solid black line) or the robust smoothing (dashed grey line) method. The length of the signal *T*_0_[*s*] = *N* ⋅ *T*, where *T* = 1 sample step (bin size) and *N* is the number of signal samples.

The trend *t*[*n*] has to be estimated from *x*[*n*] using a suitable method. Two types of frequently used detrending methods are investigated, specifically smoothing and robust smoothing approximation. Smoothing approximation exploits one or more Gaussian or exponential functions; their number or type depends on the shape of the time series. A method exploiting two Gaussian fittings is chosen according to the character of the experimental nonstationary neutrophil signals used in this paper; the robust smoothing method is based on the cosine transform and weighting of outliers designed by Damien Garcia [10]. Both detrending methods are also used for stationary signals to assess their suitability for usage on stationary Poisson data. The difference between trends estimated by the two Gaussian fitting method (solid black line) and the robust smoothing method (dashed gray line) is illustrated on the experimental nonstationary signal from neutrophils in Fig 3.

### Data

Experimental time series and model data are used for the evaluation of the suggested PP method. Three types of experimental data are investigated in total: i) nonstationary luminol-chemiluminescence signals of human neutrophils induced by Phorbol 12 myristate 13-acetate (PMA, Sigma-Aldrich, USA) [25], ii) stationary signals of endogenous biological chemiluminescence from mung seeds [26], and iii) noise (dark count) from a photomultiplier tube (PMT) detector module. The experimental data were obtained using a selected low-noise PMT module H7360-01 (Hamamatsu Photonics K.K.) operated in a photon-counting mode (dark count with stable value of cca. 13 counts per second) in a light-tight chamber (custom-made by the Bioelectrodynamics research team, Institute of Photonics and Electronics of the Czech Academy of Sciences). These discrete-time data are obtained by accumulation of photocounts (detected photons + detector generated dark counts) in each selected time step (bin size). The bins size was 1 s and 50 ms for mung signals and neutrophil signals respectively. In order to statistically evaluate and verify the suggested PP method, the model data are used. The computer-generated model signals (denoted as model neutrophil signals hereafter) matched to the experimental neutrophil signals are generated as random signals with Poisson distribution with $\lambda \left[n\right]=\hat{t}\left[n\right]$, which is estimated from 10 realizations of experimental nonstationary neutrophil signals using the two fitting methods. Model signals of mungs are generated as random signals with a Poisson distribution with $\lambda =\hat{\mu }$ estimated from 10 realization of the experimental signals of mungs, respectively. One hundred realizations of the model signals are generated from one estimation of the trend $\hat{t}\left[n\right]$ from the neutrophil signal for each type of detrending method or mean value $\hat{\mu }$ from mungs. This means that 1000 Poisson model signals of one data type are used for evaluation of the PP method.

### Biological sample preparation

Mung seeds (*Vigna radiata*, BIO Mungs, CZ-BIO-001) were surface-sterilized with 70% ethanol (1 min) and 50% disinfecting agent (SAVO, CZ) (10 min). After sterilization the seeds were washed with distilled water 6 times and soaked for 6 h (shaken every half an hour). Then, the seeds were germinated in dark conditions on large Petri dishes with ultra-pure water. Before measurement the green covers of the seeds were removed. Totally twelve seeds were measured on the Petri dishes (5 cm in diameter).

The neutrophils suspension was isolated from venous blood of healthy donors. 12 mL of blood was taken from each donor and delivered in vacuum tubes with lithium heparin from the Institute of Hematology and Blood Transfusion in Prague (Czechia). The density gradient method [27] [28] [29] was used for isolation of neutrophils. Three different layers of liquids were placed to 15 mL plastic test tube (P-Lab, type K081151, Prague, Czechia). The bottom layer was 3 mL of histopaque solution 1119 (Sigma-Aldrich), the middle was 3 mL of histopaque solution 1007 (Sigma-Aldrich) and upper was 6 mL of whole blood. The tube was centrifuged at 890 g for 30 min at 20°C. Then, the neutrophils were removed and doubled in volume using PBS (Phosphate buffered saline). The neutrophils suspension was centrifuged at 870 g for 5 min at 4°C. The supernatant was taken off. 3 mL of lysis solution (composed of 154.4 mM ammonium chloride, 7.2 mM potassium carbonate, 126 *μ*M EDTA (Ethylenediaminetetraacetic acid), pH 7.2–7.4 [30] [31]) was added and the tube was kept for 15 min in the dark at room temperature for red blood cells lysing process. After that, 3 mL of PBS was added to the tube and another centrifugation at 870g for 5 min at 4°C took place. The supernatant was taken off. The final cell suspension were neutrophils in 2 mL of PBS with Ca^2+^ and Mg^2+^. The luminol at the final concentration of 5.6 *μ*M was used added as a chemiluminescent probe. Phorbol 12-myristate 13-acetate (PMA, Sigma-Aldrich, USA) was used to stimulate oxidative burst at the final concentration of 8 *μ*M.

### Evaluation of pre-processing method

Time domain parameters and phase space (*x*[*n*], *x*[*n* + 1]) are used for verification of the PP method. To demonstrate the effect of the PP method on the parameters used for the analysis of experimental luminescence signals, we chose the Fano factor [32], the Hurst exponent [33] computed by Rescaled Range Analysis (RRA [34, 35]) and Detrended Fluctuation Analysis (DFA [36–39]). Fano factor theory states that a Poisson process should have a value of 1 [32]. The Hurst exponent varies within the range from 0 to 1. A Hurst exponent close to 0.5 indicates a random (i.e. a stochastic) process. If it is higher than 0.5, the increments of the process are positively correlated (persistent), or conversely if it is lower than 0.5, the increments of the process are negatively correlated (anti-persistent). All analyzes and generation of model data was performed in Matlab (version R2015a, MathWorks). Below, we compare the raw, detrended, and pre-processed signals. Two types of detrending methods are used: detrending (*x*[*n*] − *t*[*n*]) and detrending+DC (*x*[*n*] − *t*[*n*] + *t*′), where DC is constant value *t*′. The use of the detrending+DC method is necessary for calculation and comparison of the results of the distribution and Fano factor analysis or for illustration of the results in segmentation analysis. For a clear graphical interpretation, in the current paper we chose *t*′ = *min*(*t*), which obeys the condition (11).

Let us summarize the original moments of the Poisson distribution of the signal *x*[*n*]. The mean at the time instant *n* is *μ*[*n*] = *t*[*n*], the variance *Var*[*x*[*n*]] = *t*[*n*], the skewness ${\tilde{\mu }}_{3}=\frac{1}{\sqrt{t[n]}}$, and the kurtosis $e_{4}=\frac{1}{t[n]}$. Eq (9) gives the following moments of Poisson distribution of the signal *s*[*n*]: *μ*[*n*] = 0, the variance *Var*[*s*[*n*]] = 1, while the skewness and the kurtosis are unchanged. But Eq (10), which involves both the signal trend shift and the quantization, introduces some changes and errors we analyze in the following text. First, Eq (10) without the quantization gives following moments: *μ*[*n*] = *t*′[*n*] which is constant, the variance is also equal to *t*′[*n*], the skewness ${\tilde{\mu }}_{3}=\frac{1}{\sqrt{t^{\prime }\left[n\right]}}$, and the kurtosis $e_{4}=\frac{1}{t^{\prime }\left[n\right]}$. Eq (11) suggests that the stationary trend *t*′[*n*] might be less than the original nonstationary trend *t*[*n*]. Second, the nonlinear operation represented by the quantization clearly introduces a certain bias and variance into the transformed data and into their statistical moments. As a result, the moments of the signal *p*[*n*] including the skewness or kurtosis are not reproduced faithfully to a full extent. To quantitatively assess the influence of the suggested signal transformation and quantization given by Eqs (9)–(11) on the final result the signal-to-noise ratio (*SNR*) using the mean square value [40] can be used as a measure

$$\begin{array}{c} SNR=10\log\frac{P_{sig}}{P_{noise}}. \end{array}$$ (12)

*P*_*sig*_ and *P*_*noise*_ are the signal power (mean square value) and the noise power, respectively. The Poisson distribution implies that the signal power is *P*_*sig*_ = *t*′ + (*t*′)^2^ (we omit the index *n* for simplicity). The same holds for the measurement noise power *P*_*noise*_. Both, the original and transformed signal (and the noise) samples are the integer numbers thus the quantization step size Δ is equal to 1. Then the quantization noise with the uniform distribution has the power *P*_*noiseQ*_ = 1/12 [40]. The resulting signal-to-noise ratio caused by the quantization is then

$$\begin{array}{c} SNR_{Q}=10\log\frac{P_{sig}}{P_{noiseQ}}=10\log\frac{\left(t^{\prime }+{\left(t^{\prime }\right)}^{2}\right)}{\frac{1}{12}}=10\log\left(12\right)+10\log\left(t^{\prime }+{\left(t^{\prime }\right)}^{2}\right). \end{array}$$ (13)

This equation enables us to estimate a range of possible values *t*′ using the information about the measured *SNR* of the respective experiment. The admissible minimum value of $t_{min}^{\prime }$ can be obtained as the number for which the level of the quantization noise is less than the level of noise of the photomultiplier tube. In other words the *SNR*_*Q*_ given by (13) and caused by the quantization process has to be greater than the measured *SNR* of a respective experiment. For example, the typical value of the *t*′ for the mung seeds experiment is $t_{sig}^{\prime }=50$ giving the signal power *P*_*sig*_ = 2550 while $t_{noise}^{\prime }=13$ gives the noise power *P*_*noise*_ = 3.5. Eq (12) returns the measured *SNR* = 12 dB while the *SNR*_*Q*_ caused by the quantization and given by Eq (13) is *SNR*_*Q*_ = 45 dB. This result clearly shows that the error caused by the quantization is much lower than the error (noise due to dark count) introduced by the PMT detector module. When one admits *SNR* ≤ *SNR*_*Q*_ then the suggested transformation (9)–(11) can be used for $t_{min}^{\prime }\approx 0.7$. The results of the neutrophil experiment are: $t_{sig}^{\prime }=700$ and $t_{noise}^{\prime }=0.65$ yields *SNR* = 60 dB, *SNR*_*Q*_ = 67 dB, and $t_{min}^{\prime }\approx 315$. On the other hand, the maximum value of *t*′ is determined by the number of samples *N* available in a respective experiment. A reasonable choice seems to be *t*′ ≤ *N*/10. In this case the quick check of data transformation (9)–(11) can be performed by the inspection of the phase space grid to see if it still has a lattice structure. For example, the maximum value of *t*′ is about 3000 for the neutrophil experiment with *N* = 30000 samples. Another problem is the bias $b\left(\hat{\Phi }\right)=E\left[\hat{\Phi }\right]-\Phi$ [40] of the skewness and kurtosis caused by the quantization process. Symbol Φ stands for the true but unknown parameter (here skewness or kurtosis) and $\hat{\Phi }$ is the respective estimate. The rough estimate of the maximum bias error can be performed as follows. As mentioned before the quantization step size Δ is equal to 1 therefore the resulting maximum error is also 1 (more precisely (-1) because rounding to the floor is used). Thus the bias can be approximately expressed as $b\left({\tilde{\mu }}_{3}\right)=\frac{1}{\sqrt{t^{\prime }-1}}-\frac{1}{\sqrt{t^{\prime }}}$ for the skewness or $b\left(e_{4}\right)=\frac{1}{t^{\prime }-1}-\frac{1}{t^{\prime }}$ for the kurtosis. The mung beens experiment with *t*′ = 50 yields $b({\tilde{\mu }}_{3})=0.0014$ and *b*(*e*_4_) = 4.10^−4^. Therefore the bias error is negligible for our experiments. But the admissible minimum value of *t*′ is not so low as reported above. First, *t*′ must be greater than 1 as suggested by equations for the bias error. Second, the bias error is large for the low values of *t*′. For example, for *t*′ = 10 is 2% which is greater than 0.14% for the mung been experiment with *t*′ = 50.

## Results and discussion

### Quality of poisson pre-processing

**Fig 4:**
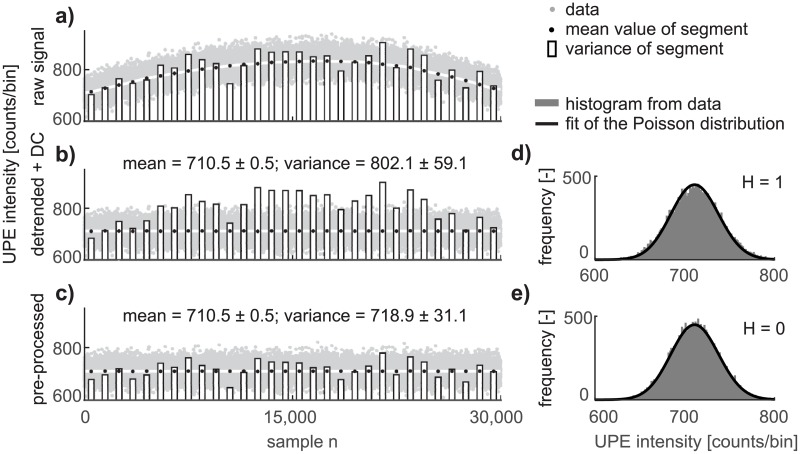
Our poisson preprocessing method recovers poisson distribution. a) The experimental signal from neutrophils, where grey dots are the number of counts per 50 ms, the white line is a trend determined by the robust smoothing method, black dots are mean values in segments, and the bars are the variance in segments. b) Detrended (DC component min(t[n] added) and c) the pre-processed signal from neutrophils. On the graphs d) and e) are histograms from the data (gray bars), and the black line is the computed Poisson distribution with the parameter λ estimated from the experimental data.

The goal of the PP method is to render the data mean and variance stationary while simultaneously preserving the original Poisson distribution. Because the mean and variance of the preprocessed signal *p*[*n*] do not change over time (they are constant), the signal *p*[*n*] can be considered as a wide-sense stationary (wss) one. In fact, wss requires that the first moment (mean) and the second moment (covariance) do not vary with respect to time. Thus to be more precise, the suggested PP ensures only trend and variance stationarity. This part is focused on the evaluation of the PP method in a time domain and in a phase space. The PP method requires trend estimation. Both types of fitting methods used give the same results, as described below. The results of the detrending and PP methods obtained by using the robust smooth fitting method are given in Fig 4.

Fig 4 compares the time and statistical parameters of the a) raw (measured), b) detrended+DC, and c) pre-processed experimental signal of neutrophils. To illustrate the differences between the detrended and pre-processed signal, the DC component is added into the detrended signal corresponding to the minimum value of the trend min(t[n]). The differences in signal shape between the detrended (Fig 4b, gray dots) and the pre-processed signal (Fig 4c, gray dots) cannot be seen by the naked eye. For the purpose of illustrative visualization of the difference between the detrending and PP methods, the following approach is applied. The signal is divided into 30 segments, each containing 1000 samples. The mean value ${\hat{\mu }}_{i}$ (black points) and variance ${\hat{\sigma }}_{i}^{2}$ (bar graph) in the *i*-th segment are calculated. The length of segments is selected as a compromise between the errors in the estimated trend $\hat{t}\left[n\right]$ (white line) and the values of the mean ${\hat{\mu }}_{i}$ (black dots), as seen in Fig 4a. The results of segment analysis show that the variance of the detrended signal is still almost the same as the variance of the raw signal (compare the bar graphs in Fig 4a and 4b), whereas the variance of the pre-processed signal corresponds to its mean value (Fig 4c, bar graphs versus black points). Our PP method ensures that the variance of the pre-processed signal is equal to its mean, in contrast to the detrended signal, whose variance differs from its mean. The deviation from equality between the parameters *μ*_*i*_ and *σ*_*i*_ (according to the equation *t*_*i*_[*n*] = Var(*x*_*i*_[*n*])) in segments of experimental or model neutrophils data (raw, pre-processed) is mainly caused by the stochastic character of the signals. Imperfect estimation of the trend, the final length of the intervals, or additive composition of the photonic signal and noise could also contribute to this deviation.

Preservation of the Poisson distribution is verified by the chi-square two-sample test [41]. The null hypothesis stating that the data come from a Poisson distribution is rejected for the detrended signal from the experimental data of neutrophils (Fig 4d) and not rejected for the pre-processed data (Fig 4e, p-values higher than 0.9). This conclusion is still valid for the model data of neutrophils (p-values typically higher than 0.9). The null hypothesis is not rejected for the stationary luminescence experimental data and model data from mung seeds before and after application of the PP method (p-values typically higher than 0.8). The null hypothesis is rejected for detector noise (which is known to be super-Poissonian [26]) before and after application of the PP method.

**Fig 5:**
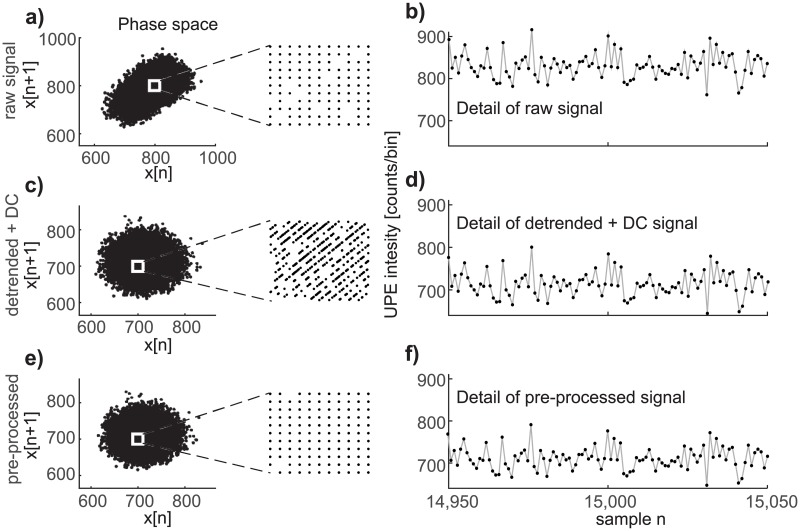
Phase space provides better assessment of the pre-processing than the time domain. a) Experimental neutrophil chemiluminescence data in the phase space and a close-up view of the raw neutrophils data in phase space, b) detail of the time series of raw experimental data from neutrophils luminescence. c) Detrended+DC data in phase space and a close-up view, d) a detail of the time series of the detrended+DC data. e) The pre-processed data in phase space and a close-up view, f) detail of the time series of the pre-processed data.

Another view of the property of the PP method is obtained from the phase space (x[n], x[n+1]). Fig 5 demonstrates the behavior of nonstationary experimental Poisson signals from neutrophils in the phase space. The almost elliptic shape of data from neutrophils in the phase space (Fig 5a) is caused by the existence of a nonstationary trend. This statement is also verified on model Poisson data of neutrophils. After detrending the experimental neutrophils data or pre-processing same data by the suggested PP method, rendering the data mean in both cases, the cluster shape in the phase space is changed from an ellipse to a circle (Fig 5c and 5e). However, on zooming in the central part of the data in the phase space, it is clearly seen that the structure of the data is different. After detrending, the dependence between adjacent samples is removed, causing changes in the structure of the lattice (compare Fig 5a and 5c). The PP method defined by (9) and (10) preserves the structure of the lattice in the phase space (see Fig 5e). The data waveform in the time domain remains almost the same, as can be seen by comparing details of the raw (Fig 5b), detrended+DC (Fig 5d), and pre-processed (Fig 5f) signals. Detrending and the PP method change the scale (energy) of the signal but the pattern of the time series is preserved. It can be concluded that while the details of the phase space representation is a very sensitive descriptor, the signal waveform itself is not a good descriptor for revealing differences between results achieved by detrending or by the PP method. Preservation of the phase space lattice by the PP method is closely connected with the fact that the PP method does not change the Poisson distribution of the data.

We also tested our PP method on stationary data with and without a Poisson distribution and nonstationary non-Poisson data. Stationary Poisson data remained unchanged, as verified on real photonic data of mungs and model data of mungs. If stationary non-Poisson data are non-integer, the PP method takes only the integer part of the data; we showed this on model data with a normal distribution (both types: *μ* = *σ*, *μ* ≠ *σ*). In the case of stationary non-Poisson integer data, the PP method does not change the data; this was verified on detector noise data. Nonstationary non-Poisson data are radically changed after using the PP method (verified on model data with a normal distribution). This conclusion is consistent with the theoretical assumption based on (9) and (10).

### Influence of poisson pre-processing method on the result of further signal analysis

Fractal analysis of photonic signals arising, for example, from chemiluminescence and fluorescence is one of several possible ways to obtain further information from photonic signal time series, offering the promise of new fingerprints and markers beyond mere intensity, optical wavelength, and simple correlation analyzes. Signals from certain luminescent systems require fractal/chaos based approaches for their analysis [42, 43]. Several authors used the Fano factor [44–46], Hurst exponent [9, 47, 48] or further advanced methods such as description in terms of quantum squeezed states [49–51] to analyze photonic data and found correlations with biological parameters. However, most of these earlier works either did not use any detrending or used just a simple subtraction of the mean value of the signals so the interpretation of their results is ambiguous [52].

**Fig 6:**
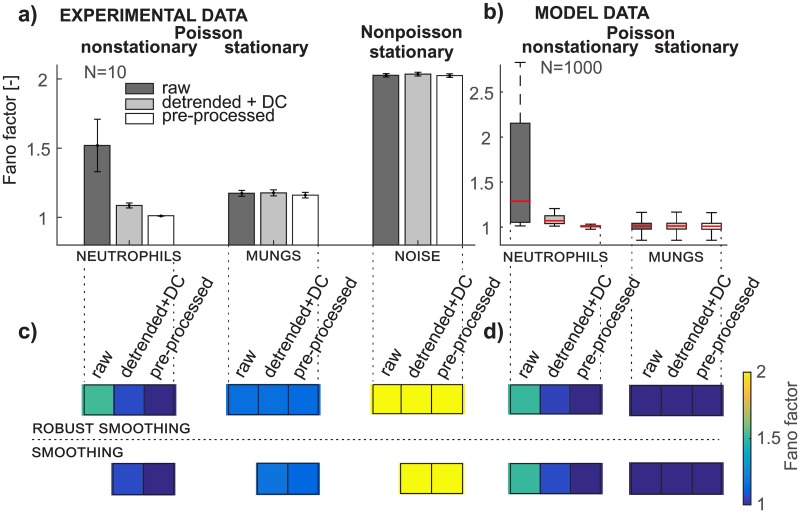
Pre-processing removes artifactual findings in fano factor analysis. Preprocessing removes artifactual findings in Fano factor analysis. a) Bar graphs depict mean values and the 95% confidence interval of the Fano factor of experimental data from neutrophils, mungs, and detector noise for all three data types (raw, detrended+DC, and pre-processed); b) the box plot depicts the distribution of the Fano factor of model data of neutrophils and mungs for all three data types (raw, detrended+DC, and pre-processed); c) the color bar represents the mean value of the Fano factor from experimental data for both types of detrending methods (smoothing, robust smoothing); d) the color bar represents the mean value of the Fano factor from model data for both types of detrending methods.

**Fig 7:**
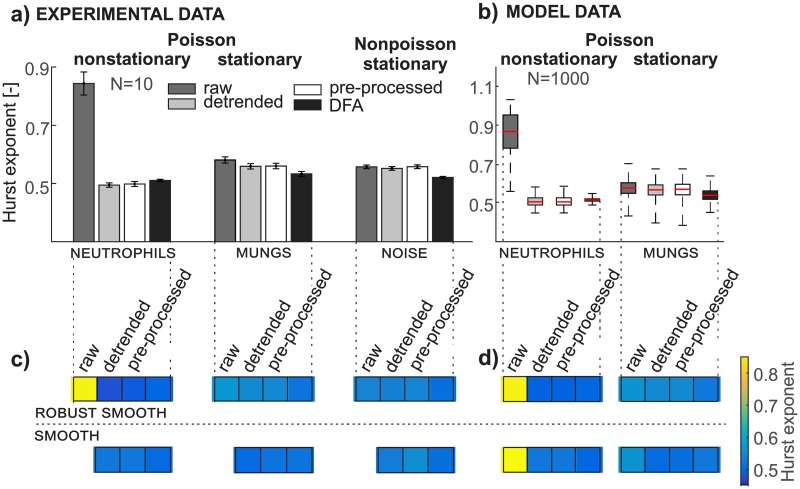
Pre-processing removes artifactual findings in hurst exponent analysis. Preprocessing removes artifactual findings in Hurst exponent analysis. a) Bar graphs depict mean values and the 95% confidence interval of the Hurst exponent of signals estimated from Rescaled range analysis and Detrended Fluctuation Analysis from experimental data of neutrophils, mungs, and detector noise for all three data types (raw, detrended and pre-processed); b) the box plot depicts the distribution of Hurst exponent of signals from model data of neutrophils and mungs for all three data types (raw, detrended, and pre-processed); c) the color bar represents the mean value of the Hurst exponent from experimental data for both types of detrending methods (smoothing, robust smoothing); d) the color bar represents the mean value of the Hurst exponent from model data for both types of detrending methods.

Here we demonstrate that our PP method removes artifactual findings from Fano factor (Fig 6) or Hurst exponent (Fig 7) analysis of photonic signals and performs better than just detrending with an added DC component in the case of Fano factor analysis. The Fano factor and Hurst exponent estimated by RRA are sensitive to the trend in nonstationary data and thus detrending and the PP method radically change their values, as illustrated in Figs 6c, 6d, 7c and 7d for both types of neutrophils data (experimental and model). Comparison of the Fano factor from experimental and model data of neutrophils gives almost the same results (Fig 6c and 6d). This conclusion corresponds to the assumption that the nonstationary raw neutrophils data come from a Poisson distribution, which is confirmed by a chi-square two-sample test of pre-processed data with a 0.05 level of significance. The hypothesis of the Poisson distribution is rejected for experimental and model neutrophils data after detrending. The Fano factor of both types of model data (Fig 6b) after using the PP method for mungs and also for raw and detrended data is equal to the expected value of 1. The difference between the Fano factor in model and experimental data from mungs (Fig 6c and 6d) is caused by the fact that the experimental data are composed from the chemiluminescence signal and non-Poisson detector noise while the model data are not. If the SNR is low, the non-Poisson detector noise depreciates the final signal and its distribution, as we recently demonstrated [26]. Although the Fano factor of experimental mungs data after detrending and the PP method is slightly higher than 1 (specifically, it is 1.17), the chi-square two-sample test confirms the hypothesis of a Poisson distribution (summarized in the section Quality of Poisson pre-processing). The detector noise is found to be non-Poissonian since its Fano factor equals 2.02 (Fig 6a) and also chi-square two-sample test rejected the hypotheses of the Poisson distribution. Both types of detrending (smoothing and robust smoothing) leads to very similar values of the Fano factor for all data considered (Fig 6c and 6d).

RRA and DFA yield an estimate of the Hurst exponent but the principle of its calculation is different. The Hurst exponent from RRA responds to the trend (Fig 7a and 7b, neutrophils) whereas DFA is designed for nonstationary data since the detrending procedure is applied within the DFA method. However, the disadvantage of the DFA is in its subjective setting of the scale parameter for the segmentation. Thus the DFA, when applied to a nonstationary signal, could yield incorrect results for an inappropriate selection of the scale parameter.

The output from DFA shows that the results from raw, detrended, and pre-processed data are almost identical (Fig 7c and 7d, black bar). Application of the RRA to detrended or pre-processed signals provides a very similar value, which means that it is not sensitive to small changes in the variance of data applied in the PP method. This conclusion holds for both types of the model signals (Fig 7b) as well as for all three types of experimental signals (Fig 7a). The RRA of stationary signals should give the same result for the Hurst exponent estimated for raw, detrended, and pre-processed data. The noticeable exception is the Hurst exponent from the raw data of experimental and model mungs (Fig 7a and 7b) caused by detrending in the PP method, although the stationarity of the signals from mungs is verified by the Lilliefors test (level of significance = 0.05, p-values higher than 0.4). We also tested the influence of signal length (1000, 10000, and 30000 samples) on the results of RRA from raw, detrended, and pre-processed model data of mungs. The differences between the results are smaller if the length of the signal is greater. The differences in results between the raw and detrended signals of model mungs are larger than those for the raw and pre-processed signal. The Hurst exponent (RRA) from nonstationary neutrophils data shows artifactual findings of a positive correlation while the Hurst exponent from detrended, preprocessed neutrophils data, and DFA reveals the actual uncorrelated character of the data.

According to the results of the Fano factor and the Hurst exponent estimated from neutrophils data (Figs 6c, 6d, 7c and 7d) the type of detrending method (smoothing and robust smoothing) is not a crucial part of the PP method. A suitable method for trend estimation should yield a smoothed curve following slow changes in the signal.

## Conclusion

We present a new pre-processing method for nonstationary Poisson signals in this paper. The assumption of the input signal properties is that its mean is equal to its variance (E[*x*[*n*]] = Var[*x*[*n*]]) and signal samples are nonnegative integers (x[n]≥0∧x[n]∈Z). Our Poisson pre-processing method renders the signal stationary and preserves the relation between the mean and variance of the random signal composed of non-negative integer samples. This property is illustrated by the segmentation analysis and verified by statistical testing. Moreover, the pre-processed signal keeps its original rectangular structure in the phase space, making our pre-processing method potentially useful for preparing the signals for further complexity and chaos-theory-based analyzes. Application of the pre-processing method to nonstationary signals that are non-Poisson never recovers a Poisson distribution, and hence *a posteriori* check of whether the analyzed signal originated from a Poisson distribution is possible. Moreover the Poisson pre-processing method does not change the distribution of stationary integer data and causes only minor changes due to rounding when applied to non-integer data such as those originating from a normal distribution.

While our primary motivation was to focus on the pre-processing and analysis of photonic signals such as bio/chemiluminescence and fluorescence, the method we developed is completely general and can be applied to any signal originating from a Poisson process. Furthermore, our method can be generalized to any mean-variance-coupled signals of non-Poisson distribution provided that the analytic formula for the dependence of the mean and the variance is known.

We believe that the application of our method can prevent artifactual findings and enable the analysis of nonstationary photonic signals that might otherwise have been unusable and discarded due to the baseline drifts.

## Supporting information

S1 Dataset Dataset contains all raw experimental and computer generated photocount signals used in this paper. (ZIP)
